# The incidence of experimental smoking in school children: an 8-year follow-up of the child and adolescent behaviors in long-term evolution (CABLE) study

**DOI:** 10.1186/1471-2458-11-844

**Published:** 2011-11-03

**Authors:** Hsing-Yi Chang, Wen-Chi Wu, Chi-Chen Wu, Jennifer Y Cheng, Baai-Shyun Hurng, Lee-Lan Yen

**Affiliations:** 1 Division of Preventive Medicine and Health Service Research, Institute of Population Health Sciences, National Health Research Institutes, Zhunan Town, Miaoli County, Taiwan, ROC; 2 School of Health Care Management, Department of Nutrition and Health Sciences, Kainan University, Taiwan, ROC; 3 Center for Population and Health Survey Research, Bureau of Health Promotion, Department of Health, Taichung, Taiwan, ROC; 4 Institute of Health Policy and Management, College of Public Health, National Taiwan University. Rm. 623, No. 17, Xu-Zhou Road, Taipei 100, Taiwan, ROC

**Keywords:** cigarette smoking, adolescent behavior, epidemiological factors, socioeconomic factors

## Abstract

**Background:**

Studies have established that most regular adult smokers become addicted in their adolescent years. We investigated the incidence of and risk factors associated with initial experimental smoking among a group of school children who were followed for 8 years.

**Methods:**

We used cohort data collected as part of the Child and Adolescent Behaviors in Long-term Evolution (CABLE) study, which selected nine elementary schools each from an urban area (Taipei City) and a rural area (Hsingchu county) in northern Taiwan. From 2002 to 2008, children were asked annually whether they had smoked in the previous year. An accelerated lifetime model with Weibull distribution was used to examine the factors associated with experimental smoking.

**Results:**

In 2001, 2686 4^th^-graders participated in the study. For each year from 2002 to 2008, their incidences of trial smoking were 3.1%, 4.0%, 2.8%, 6.0%, 5.3%, 5.0% and 6.0%, respectively. There was an increase from 7^th ^to 8^th ^grade (6.0%). Children who were males, lived in rural areas, came from single-parent families, had parents who smoked, and had peers who smoked were more likely to try smoking earlier. The influence of parents and peers on experimental smoking demonstrated gradient effects.

**Conclusions:**

This study used a cohort to examine incidence and multiple influences, including individual factors, familial factors, and community factors, on experimental smoking in adolescents. The findings fit the social ecological model, highlighting the influences of family and friends. School and community attachment were associated with experimental smoking in teenagers.

## Background

Smoking is one of the most popular forms of drug use and is a major risk factor for lung cancer and cardiovascular disease [1]. In a systematic review of 17 studies, smoking was found to be associated with peripheral arterial disease not only among current smokers but also among former smokers [2]. Due to its public accessibility, smoking has become one of the world's most prominent public health issues and is a leading cause of premature death worldwide [3,4]. A high prevalence of smoking has been observed in Chinese societies [5]. Several studies have established that most regular adult smokers become addicted to nicotine in their adolescent years [6-8]. Public health researchers have conducted extensive research on adolescent smoking and its relationship with factors such as parental and peer relations, academic performance, socioeconomic factors, and environmental factors [9]. Due to the specific targeting of advertising by the tobacco industry, growing populations of adolescents in both developing and developed countries are taking up smoking [10]. If not effectively addressed, the world will see generations of young smokers suffer from highly preventable cardiovascular diseases and risk premature death. The reasons behind adolescent smoking are complex and involve multiple influences, including individual factors, socioeconomic factors, familial factors, peer influences, school and environmental factors. It has been reported that the majority of youth experimental smokers become daily smokers [7,8]. An understanding of the age of experimental smoking and related risk factors is important for designing early intervention programs targeting high-risk groups.

Many studies have monitored the smoking status of youth worldwide. The Global Youth Tobacco Survey (GYTS) is the largest surveillance system, which conducts periodic surveys on tobacco use in a wide range of countries [11]. Many longitudinal studies have also explored the factors predicting regular smoking in teens [8]. However, these studies, especially longitudinal studies of experimental smoking in school children, have rarely been conducted in Asia. Previous studies examined either current smoking behavior or the initiation age of smoking. As mentioned above, a large proportion of experimental youth smokers continue to smoke in adulthood. It is important to understand when these youth first tried smoking and the factors that are associated with these trials.

The Child and Adolescent Behaviors in Long-term Evolution (CABLE) study [12] was initiated to examine the behavioral development of school children in Taiwan. Children were visited annually and were asked questions related to their behaviors. The study has accumulated long-term information on these children's behaviors and risk factors. Information concerning when children first tried smoking and the factors associated with smoking experimentation can be extracted from these data. The purpose of this study was to investigate the incidence of experimental smoking and its risk factors in a group of school children who were followed for 8 years.

## Methods

Data for this study came from the study of Child and Adolescent Behaviors in Long-term Evolution (CABLE) [12,13]. The study was designed to observe the development of children based on the ecological model, which emphasizes that individual, interpersonal, organizational, community and public policy factors shape the development of a child. Additional information was collected during the yearly follow-ups to reflect problems faced by the children at that age, but the changes on questions were minor. Students from urban and rural areas were sampled to identify the developmental differences between these two groups [13]. Nine elementary schools each from an urban area (Taipei City) and a rural area (Hsingchu County) in northern Taiwan were selected. The 1^st ^and 4^th ^graders in each school (representing the 1^st ^and 2^nd ^cohorts) were selected as the baseline cohorts to be followed annually. The CABLE study was approved by the Internal Review Board of the National Health Research Institutes (approval code: EC9009003). All parents of students in the study signed a consent form allowing their children to participate. We used data from the initial cohort of fourth-graders in this study because they were entering adolescence. Complete data on all 8 years of follow-up were available for more than two-thirds of the children.

The outcome variable was experimental smoking from 2002 to 2008. Each year, children were asked whether they had smoked in the past year. We asked the question, 'Have you smoked cigarettes, even one puff?' The answers included (1) never; (2) not this year, but the year before; (3) not this month, but this year; (4) one or two times this month; (5) many times this month; and (6) almost every day this month. This set of questions was similar to the Global Youth Tobacco Survey [14]. Students who had not answered 'yes' in previous years and who answered 'yes' in a subsequent year were considered new cases. Because we were studying experimental smoking in children aged approximately 10 years, we considered one inhalation of tobacco to be an incidence of experimental smoking. In addition to individual factors, we were interested in the effects of family and community on the initiation of smoking.

Individual factors included gender, residential area, self-perceived school performance, depressive symptoms and self-competence. Depressive symptoms were measured by 7 questions, which asked about loss of appetite, feeling sad, crying for no reason, getting upset over nothing, feeling frightened, difficulty sleeping, and lack of motivation to do anything in the previous two weeks. A three-point-scale was used with 1 point given for not at all, 2 for sometimes, and 3 for often. The sum of the scores for all items was used. A higher score implied more depressive symptoms. The questions were based on the depressive symptoms of the Center for Epidemiological Studies Depression Scale for Children (CES-DC) [15], which has been widely used in Taiwanese surveys. Cronbach's α, which measures how well each individual item in a scale correlates with the sum of the remaining items, was used for the internal consistency or validity of the scales. The survey of 8^th ^graders was used to estimate Cronbach's α. The widely accepted social science cut-off is an alpha of .70 or higher for a set of items to be considered a scale [16]. The Cronbach's α was 0.74.

Self-competence was measured by 6 items, including optimism, feeling happy, willingness to try new things, working hard, facing problems positively, and perceiving oneself as being as good as others. The concept was based on Tafarodi and Swann [17]. The Chinese version was evaluated by Song et al. [18]. A 5-point Likert scale was used, with scores ranging from 1 (never) to 5 (always). The scores of the 8^th ^graders were used to calculate Cronbach's α, which was 0.79.

Family factors included socio-economic status, coming from a single-parent family and the degree of parental support and supervision. Parental smoking was also assessed. Family socioeconomic status was measured by family income and father's education level. The categories of monthly family income were (1) less than 19, 999NTD (new Taiwan dollars, 1 NTD ≈0.03 USD); (2) 20,000~39, 999NTD; (3) 40, 000NTD~59, 999NTD; (4) 60,000~79, 999NTD; (5) 80,000~99, 999NTD; (6) 100,000~119, 999NTD; (7) 120,000~139, 999NTD; and (8) ≥ 140, 000NTD. Education levels were (1) elementary school or less; (2) junior high; (3) senior high; (4) vocational school; (5) college; (6) university; and (7) graduate school or above. We added together the levels of family income and education and then divided them into 3 categories: low < 8 (1^st ^quartile); medium 8-13; and high 13 (4th quartile).

Parental smoking status was assessed for both parents. The combination of answers resulted in three categories: (1) both parents smoke; (2) only one parent smokes; and (3) neither parent smokes. The questions on parental support and supervision were part of family interactions [19]. They were developed by a panel of experts specializing in behavioral sciences and education. They adapted the concepts from various researchers [20,21]. Once the experts reached consensus, the questions were examined by the fieldworkers to evaluate their feasibility. Then, the CABLE team has extracted factors on family interactions. The family interactions consisted of 6 aspects, including parental support, family activities, psychological control, parental discipline, behavioral supervision, and family conflict [19]. We only used two aspects in this study, which were parental support and supervision. Parental support was assessed by 6 questions: providing encouragement in difficult times, praising good performance, providing comfort when you are upset, taking care of you when you are sick, listening, and taking an interest in your school life. Each response had four options ranging from 1 (never) to 4 (every time). Scores for each item were added together to obtain the score for parental support. The Cronbach's α was 0.91. Parental supervision was assessed by four questions, which asked whether parents were aware of what children did in their spare time, what they did on their way home from school, who they spent time with, and how they used their allowance. A 4-point scale was used, with 1 indicating that parents did not know anything and 4 indicating that they knew everything. Scores for each item were summed to obtain the score for parental supervision. Cronbach's α was 0.77. Details of the questions can be found in Appendix 1.

Peer smoking, school attachment and neighbors were considered as community factors. Children were asked whether none, a few, about half, most or all of their peers smoked. School attachment was assessed by whether children liked their current school, teachers, and classmates. Responses to each item had five options ranging from 1 (do not like it/them at all) to 5 (like it/them very much). These questions were included in the questionnaire from 2003. The average of scores from 2003 to 2008 was used in the analysis. Eight items were used to assess community attachment: (1) Do you or your family visit or talk to your neighbors? (2) When you go away, does your family ask neighbors to house sit, including checking the mail, watering the garden, and feeding the dog? (3) Do you think your neighbors are trustworthy? (4) Do you think your neighbors are kind and friendly? (5) Do you and your family participate in community activities? (6) Do you like where you live? (7) Do you think your living environment is safe? (8) Are there ever strange people wandering around the neighborhood? A 5-point scale was used for each item, with 1 point indicating never and 5 points indicating all the time. A score in the lowest quartile (22 points) was considered low, and a score in the upper quartile (29 points) was considered high. Details of the questions used in all scales are given in Appendix 1 (Additional file 1, appendix 1).

The event time was 4^th ^grade to the year of experimental smoking. Because experimental smoking could occur before the first interview, between the two interview years, or after 2008, the data contained all types of censoring: left censoring, interval censoring and right censoring. The survival time was the time that students never smoked. A parametric model is appropriate for this type of data. A Weibull distribution consists of two parameters describing the shape and scale of the distribution curve [22]. Therefore, an accelerated lifetime model (ALT), which models the survival time assuming Weibull distribution, was used. All analyses were conducted using SAS version 10 (SAS Inc., Cary, USA).

## Results

**Figure 1:**
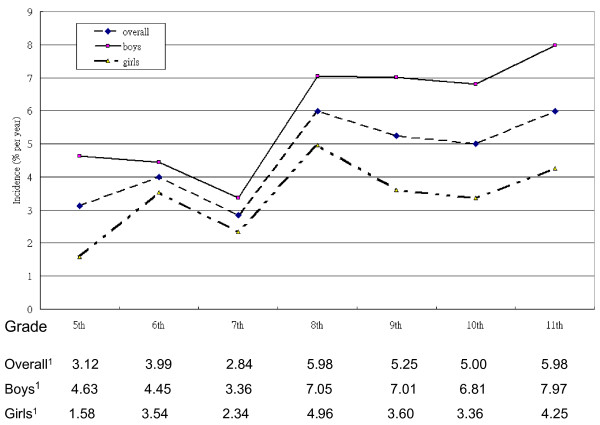
**Incidence (1/100) of smoking in school children from 2002 to 2008**. ^1^. Incidence rate (1/100)

Table 1 shows the characteristics of the study subjects. There were 2071 subjects who participated in the study in 2001. In 2002, we sent invitations to those who did not consent in 2001, and 615 of these subjects re-joined the study. Therefore, the total number of children at the start of the study was 2686 4^th^-graders. Over two-thirds of the participants were followed for 8 years. More boys than girls had tried smoking at least once during the 8-year follow-up. More ever-smokers lived in a rural area (Hsinchu) than in an urban area (Taipei city). Children who had poorer perceived school performance were more likely to start smoking during the follow-up. Children with medium to low socioeconomic status were more likely to smoke than those with high socioeconomic status. Children with one or both parents who were smokers were more likely to be smokers themselves. Socioeconomic status was inversely associated with smoking initiation, as was community attachment. All of these factors reached statistical significance. Children who started smoking had significantly higher depressive symptom scores, lower self-competence, lower parental support, less parental supervision, and lower school attachment scores. The smoking incidence rate is shown graphically in Figure 1. There was a drop in incidence in the 7^th ^grade followed by a sharp increase in the 8^th ^grade. Boys were consistently more likely to take up smoking than girls.

**Table 1:** Characteristics of the study sample

	Never smoked		Smoked			
	
Characteristic	n	(%)	n	(%)	*χ^2^*	
Gender (n = 2686)					78.21	***
Boys	831	(60.13)	551	(39.87)		
Girls	992	(76.07)	312	(23.93)		
Residential area (n = 2686)					43.88	***
Taipei (urban)	1062	(73.39)	385	(26.61)		
Hsinchu (rural)	761	(61.42)	478	(38.58)		
Perceived school performance (n = 2341)					6.46	*
Poor	861	(67.21)	420	(32.79)		
Fair	575	(64.90)	311	(35.10)		
Good	130	(74.71)	44	(25.29)		
Single-parent family (n = 2668)					54.92	***
No	1559	(71.03)	636	(28.97)		
Yes	253	(53.49)	220	(46.51)		
SES (n = 2611)					54.18	***
Low	341	(57.02)	257	(42.98)		
Medium	1037	(69.41)	457	(30.59)		
High	400	(77.07)	119	(22.93)		
Parental smoking (n = 2418)					84.48	***
Neither	743	(78.05)	209	(21.95)		
One	815	(64.43)	450	(35.57)		
Both	99	(49.25)	102	(50.75)		
Peer smoking (n = 2540)					463.45	***
None	556	(90.11)	61	(9.89)		
Few	900	(71.60)	357	(28.40)		
Over half	234	(35.14)	432	(64.86)		
Community attachment (n = 2686)					12.71	**
Low	591	(66.18)	302	(33.82)		
Medium	893	(66.59)	448	(33.41)		
High	339	(75.00)	113	(25.00)		

Scores	Mean	(SD)	Mean	(SD)	*t*	

Depressive symptoms	11.06	(2.76)	11.57	(2.79)	-4.46	***
Self-competence	21.15	(4.01)	20.28	(3.97)	5.13	***
Parental support	18.89	(4.32)	17.29	(4.71)	8.41	***
Parental supervision	13.22	(2.82)	12.21	(3.19)	7.74	***
School attachment	10.86	(1.43)	10.22	(1.54)	10.18	***

*: *p *< 0.05; **: *p *< 0.01; ***: *p *< 0.001

Table 2 shows the results of survival analysis. We used an accelerated failure time model assuming a Weibull distribution. The scale was 0.57, which indicated that the hazard was increasing at a decreasing rate. A negative value indicated an early initiation. Boys and students who lived in a rural area, came from a single-parent family, had parents who smoked, had peers who smoked, had low school attachment, or had median community attachment started smoking earlier than did their counterparts.

**Table 2:** Relative risks for smoking initiation in school children

Variable	Estimate	s. e	**95% C.I**.	χ^2^	Pr > χ^2^
Intercept	2.56	0.34	(1.89, 3.22)	56.80	< .0001
Individual factors					
Boys vs. girls	-0.31	0.05	(-0.41, -0.20)	34.06	< .0001
Taipei vs. Hsinchu	0.23	0.05	(0.13, 0.34)	19.41	< .0001
School achievement:					
Poor vs. good	0.07	0.11	(-0.14, 0.28)	0.39	0.53
Fair vs. good	0.03	0.11	(-0.18, 0.24)	0.08	0.78
Depressive symptoms score	0.00	0.01	(-0.02, 0.02)	0.05	0.82
Self-competence	0.00	0.01	(-0.01, 0.01)	0.02	0.90
Familial factors					
Single-parent family	-0.20	0.06	(-0.32, -0.08)	10.04	0.00
SES:					
Low vs. high	-0.09	0.08	(-0.26, 0.07)	1.23	0.27
Medium vs. high	0.06	0.07	(-0.08, 0.21)	0.73	0.39
Parental smoking:					
One vs. none	-0.24	0.06	(-0.36, -0.13)	17.82	< .0001
Both vs. none	-0.37	0.09	(-0.55, -0.20)	18.07	< .0001
Parental support	0.01	0.01	(-0.00, 0.02)	1.35	0.25
Parental supervision	0.01	0.01	(0.00, 0.03)	2.22	0.14
Community factors					
Peer smoking:					
Few vs. none	-0.78	0.11	(-1.00, -0.56)	48.64	< .0001
Over half vs. none	-1.35	0.12	(-1.58, -1.12)	131.87	< .0001
School attachment	0.09	0.02	(0.05, 0.12)	20.01	< .0001
Community attachment:					
Low vs. high	-0.14	0.08	(-0.30, 0.03)	2.69	0.10
Medium vs. high	-0.15	0.07	(-0.29, -0.01)	4.15	0.04

Scale	0.57	0.02	(0.53, 0.62)		
Weibull Shape	1.75	0.07	(1.62, 1.89)		

## Discussion

This study followed a group of 4^th^-graders annually and assessed the incidence of experimental smoking and related risk factors. The results showed that the incidence of experimental smoking increased with age. There was a drop in incidence in the 7^th ^grade followed by an increase in the 8^th ^grade. Individual factors, such as gender and residential area, familial factors such as a single-parent family and parental smoking status, and community factors, such as peer smoking status, school attachment and community cohesion, were risk factors for smoking initiation in school children.

The reasons for adolescent smoking are complex and multifaceted. However, researchers have been able to detect general trends related to the commencement of smoking in adolescence. In Tyas and Pederson's review [23], age was associated with an increase in smoking prevalence and initiation. Chen and colleagues reported that the mean age of initiation of use any of tobacco, alcohol, or illicit drugs was 16 to 18 years [24]. We observed a similar pattern, except for the decrease in the 7^th ^grade, which may have been a result of the transition involved in attending a new school. In Taiwan, six years of education was compulsory until 1968 after which compulsory education was extended to 9 years. All students attend elementary school for 6 years after which they move to junior high school for the next 3 years. During the first year in a new school, students might go through a period of adjustment. This is also the time when students are entering adolescence and when they begin to explore and experiment with new things. Once they have adjusted to the new environment, the students may begin experimenting. This phenomenon could be the reason for a drop in experimental smoking incidence in the 7^th ^grade followed by a sharp increase in the 8^th ^grade. One of the advantages of our study was the examination of changes over time.

**Figure 2:**
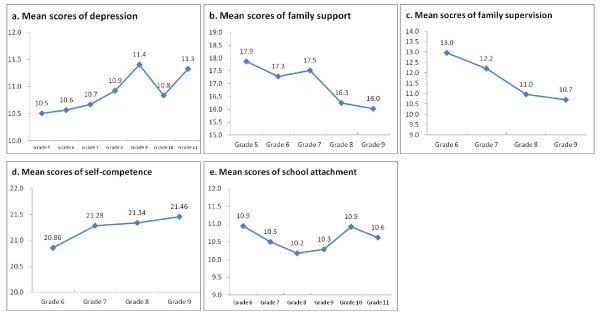
**Plots of (a) mean scores of depression; (b) mean scores of family support; (c) mean scores of family supervision; (d) mean scores of self-competence; and (e) mean scores of school attachment over time**. All values reached statistical significance at the 5% level for testing time trend.

We plotted the mean scores of depression, family support, family supervision, self-competence, and school attachment (Figure 2). The time effect for each of these repeated measurements was significant. From the plots, we see that the depression score increased over time. Family support dropped sharply between the 7^th ^and 8^th ^grades, as did family supervision. The differences between the self-competence and school attachment scores of 7^th ^and 8^th ^graders were not as significant. We speculate that family support or supervision played an important role in the sharp increase in experimental smoking. This is a difficult stage for families with adolescents. On one hand, adolescents ask for independence; on the other hand, they may not have good self-control or make good behavioral choices.

A gender difference was observed in this study. Gender differences in adolescent smoking have been discussed previously. Simons-Morton et al. [9] proposed an independent association between smoking and peer influence in boys and an association between smoking and self-control problems in girls. Societal influences are another possible contributing factor. Chinese culture still believes that males should be strong and adventurous, whereas female smoking is not socially acceptable. Our results are consistent with other observations in Taiwan [25,26].

Family structure and socioeconomic factors both play pivotal roles in the smoking status of adolescents. We found that children from single-parent families experimented with smoking earlier than their counterparts. Lin et al. reported that parents' marital status was significantly associated with adolescent smoking behavior in southern Taiwan [27]. Griesbach et al. [28] found that single-parent families and stepfamilies had a higher probability of having smokers in the household, and Covey and Tan [29] confirmed that two-parent families were protective against adolescent smoking. Painful disruptions to normal family life, such as parental separation, can trigger depressive symptoms in adolescents, leading to problem behaviors, such as smoking [29].

We found that parental smoking was a risk factor for smoking initiation in adolescents. Similar results were reported from Hong Kong [30]. There was also a possible gradient effect: children who had two parents who smoked were more likely to initiate smoking than were those with only one parent who smoked. Parental influence may play a role in adolescents' "preparatory period" during which their opinions and beliefs towards smoking are formed. This period determines their "susceptibility" to smoking, with "non-susceptible" defined as "strong negative intentions against future smoking" and "susceptible" defined as "one or more questions without strong negative intentions against future smoking" [31]. Never-smokers who believed that their parents would disapprove of their smoking in the future were less susceptible to initiating smoking. Distefan and colleagues [31] found that maternal smoking strongly encourages the progression of both male and female experimenters to become established smokers. These authors also showed that parents may serve as an impediment to adolescents' choice of friends who smoke [31]. This result explains our findings of the effect of parental smoking and lack of supervision on children's experimental smoking. However, a comparison of the influence of fathers and mothers was not the focus of our study.

The influence of peers is one of the most determinative factors in adolescents' experimental smoking. A similar factor was reported in a cross-sectional study in southern Taiwan [27]. We observed a gradient effect for peer influences. Students with a few peers who smoked were likely to experiment with smoking early but not as early as students who had a majority of peers who smoked (-0.78 vs. -1.35). All of these results reached statistical significance. However, students who smoke may over report the number of their smoking peers. In a study of Chinese adolescents and their perceived smoking norms, Chen et al. found that adolescent smokers were inclined to overestimate the number of smokers among their peers and surrounding adults [32]. The results found a positive association between perceived smoking norms and an increased risk of smoking initiation, experimentation, and establishment. In addition to educating youth about the negative consequences of smoking, it has been suggested that future anti-smoking health programs should be geared toward teaching adolescents how to overcome peer pressure to conform [33].

School is part of adolescents' community. We found that as students' attachment to school increased, they were less likely to start smoking. Adolescents spend the majority of their time at school. In addition to taking classes, adolescents develop relationships with their classmates and establish peer groups that greatly impact their decisions and beliefs. Many studies have shown that students who excel and adhere to high academic standards are less likely to engage in smoking behaviors [6,23]. In addition to students' attitudes toward studying, the school atmosphere can influence adolescent smoking status. Alexander et al. found an increase in smoking rates among popular students in schools with a higher smoking prevalence, whereas a decrease in smoking rates was evident among popular students in schools with a lower smoking prevalence [34]. We found that students with median community attachment experimented with smoking earlier than those with high attachment. However, students with low attachment did not differ from those with high attachment. It is likely that students who do not attach to the community are less likely to be influenced by the community. Further investigation on the quality of the community would provide useful information to explain these findings.

The main limitation of this study was that students were recruited from only two areas in Taiwan. Therefore, the results should be generalized with caution. Nevertheless, we obtained results similar to other studies, suggesting that the risk factors for experimental smoking are universal. Our findings should be taken into account when designing anti-smoking policies for teenagers. Another limitation of this study was the use of self-reported status. Our study was conducted in schools, and it is possible that students did not respond truthfully regarding their smoking behavior for fear of being identified or punished. Many steps were taken to ensure the students' comfort and willingness to respond truthfully. We stressed that their answers during the survey would not be provided to school teachers, and we sent our own interviewers to distribute and collect the questionnaires to ensure that teachers would not obtain the results.

## Conclusions

This study used a cohort to examine the incidence and multiple influences, including individual factors, familial factors, and community factors, on experimental smoking in adolescents. The findings presented in this study are consistent with those of other studies, particularly in terms of the association between the influences of family and friends and school and community attachment with experimental smoking in teenagers. We found gradient effects for the influence of parents and friends on the decision to experiment with smoking. We observed a sharp increase in incidence from 7^th ^to 8^th ^grades. Possible reasons were curiosity on smoking and decreasing parental support and supervision.

## Competing interests

The authors declare that they have no competing interests.

## Authors' contributions

HYC constructed the study idea, wrote the manuscript, and revised it. WCW analyzed the data and contributed intensive discussion on the study. CCW collected, edited and managed the data. JYC reviewed the literature and drafted part of the manuscript. BSH was responsible for the fieldwork and contributed intensive discussion on the study. LLY designed and initiated the CABLE study and contributed intensive discussion on the manuscript. All authors read and approved the final manuscript.

## Pre-publication history

The pre-publication history for this paper can be accessed here:

http://www.biomedcentral.com/1471-2458/11/844/prepub

## Supplementary Material

Additional file 1 **Appendix 1. Definition of explanatory variables**. Click here for file
