# Supplementary material for: The incidence of experimental smoking in school children: an 8-year follow-up of the child and adolescent behaviors in long-term evolution (CABLE) study
Source: BMC Public Health. 2011 Nov 3;11:844. doi: 10.1186/1471-2458-11-844 (PMC3229590; doi:10.1186/1471-2458-11-844)
Supplement: Additional file 1 — Appendix 1. Definition of explanatory variables. [file 1471-2458-11-844-S1.DOC]

Appendix 1.Definition of explanatory variables

| Variable | Question | Choice | Scale/re-grouping | Notes |
| --- | --- | --- | --- | --- |
| Individual factors |  |  |  |  |
| Gender | Sex | Male/female |  |  |
| Residential area | Where do you live? | Taipei/Hsinchu |  | Reference: Hsinchu |
| Self-Perceived school performance | Are you satisfied with your school performance? | 1: not satisfied at all, 2: not very satisfied, 3: fair, 4: satisfied, 5:very satisfied | Regrouped as: 1,2 poor; 3, fair; 4, 5 good | Reference: good |
| Depressive symptoms (8 items) | In the past 2 weeks, have you lost your appetite, even for your favorite food?  In the past 2 weeks, have you felt sad or been in a bad mood?  In the past 2 weeks, have you cried for no reason?  In the past 2 weeks, have you gotten upset over nothing?  In the past 2 weeks, have you felt frightened?  In the past 2 weeks, have you had trouble sleeping?  In the past 2 weeks, have you lacked motivation to do things? | 1: never; 2: seldom; 3: sometimes; 4: often; 5: always | Total score | Continuous variable, Data from 2001 was used. Missing data was imputed from the data of the following year |
| Self-competence (6 items) | Do you feel you are an optimistic person?  Do you like to try new things?  In general, do you feel you are a happy person?  Do you think you are as good as others?  Do you always try hard at everything?  When facing problems, do you try to solve them by yourself first? | 1: never; 2: seldom; 3: sometimes; 4: often; 5: always | Total score | Continuous variable, Data from 2003 was used. Missing data was imputed from the data of the following year. |
| Family factors |  |  |  |  |
| Single parent | What is your marital status? (to parents)  What is your parents’ marital status? (to children) |  |  | Single parent or not. Data from questionnaire to parents was used. |
| Family income | What is your average monthly income? (to parents) (including salary, agricultural produce, investments, bonuses, and awards) | 1: ≤19,999NTD^1^  2: 20,000-39,999NTD  3: 40,000-59,999NTD  4: 60,000-79,999NTD  5: 80,000-99,999NTD  6: 100,000-119,000NTD  7:120,000-139,999NTD  8:140,000-159,999NTD  9: >160,000NTD | Socioeconomic status (SES) combined the income scale and the education scale. | Below the lowest quartile (8) was defined as ‘low’, the upper quartile (13) was defined as ‘high’; and values between these two quartiles were defined as ‘medium’. The highest level in the study period was used as reference. |
| Father’s education | What is the highest education level of your father? | 1: elementary school or less  2: junior high  3: senior high  4: vocational school  5: college  6: university  7: graduate school |  | The highest level in the study period was used as reference. |
| Parental smoking | Do you smoke? (to parents) |  | Yes/no | Any ‘yes’ in the study period was counted as ‘yes’. |
| Parental support | Do your parents encourage you when you are having trouble doing something?  Do your parents praise you when you are a good boy/girl?  Do your parents comfort you when you are in a bad mood?  Do your parents look after you when you are sick?  Do your parents listen to you when you have something to say?  Do your parents take an interest in your school life? | 1: never; 2: once or twice; 3: many times; 4: always | Total score | Continuous variable. Data from 2001 was used. Missing data was imputed by the data of the following year |
| Parental supervision | Do your parents know what you do in your spare time?  Do your parents know what you do on the way home from school?  Do your parents know who you are with most of the time?  Do your parents know how you spend your pocket money? | 1: not at all; 2: a little bit 3: almost everything; 4: definitely know; | Total score | Continuous variable, Data from 2003 was used. Missing data was imputed from the data of the following year |
| Community factors |  |  |  |  |
| Peer smoking | How many of your friends smoked in the past year? | 1:None; 2: a few; 3: about half; 4: most 5: don’t know | Recoded 1: none; 2= a few; 3,4,5 = over half |  |
| School attachment | Do you like your current school?  Do you like your current teacher?  Do you like your current classmates? | 1: not at all; 2: a little; 3: it/they are okay; 4: I like it/them; 5: I like it/them a lot | Total score | Continuous variables. Average from 2003 to 2008 was used. |
| Community attachment | Do you and your family visit or talk to your neighbors?  When you go on a trip, does your family ask your neighbor to house sit?  Do you think your neighbors are trustworthy?  Do you think your neighbors are kind and friendly?  Do you and your family attend community activities?  Do you like where you live?  Do you feel that where you live is safe?  Are there ever strange people wandering around your neighborhood? | 5-point Likert scale | Total score | Divided into 3 groups: lowest quartile (22 points); upper quartile (>29), and anything in between was the referent group. This was asked in year 2005. |

1. New Taiwan dollars (1NTD≈0.03USD)
